# Associations between Aquaglyceroporin Gene Polymorphisms and Risk of Type 2 Diabetes Mellitus

**DOI:** 10.1155/2018/8167538

**Published:** 2018-11-27

**Authors:** Yijun Wang, Gang Chen, Qingyun Tu, Junxia Wu, Yu Qin, Zheng Zhu, Yi Shen, Li Yan, Aohan Han, Quanyong Xiang, Chun Wang

**Affiliations:** ^1^Department of Environmental Health, School of Public Health, Nantong University, Nantong, Jiangsu 226000, China; ^2^Department of Disease Control, Nanjing Jiangning District Center for Disease Control and Prevention, Nanjing, Jiangsu 211100, China; ^3^School of Public Health, Southeast University, Nanjing, Jiangsu 210009, China; ^4^Department of Comprehensive Statistics, The Third People's Hospital of Nantong, Nantong, Jiangsu 226000, China; ^5^Department of Chronic Non-Communicable Disease Control, Jiangsu Provincial Center for Disease Control and Prevention, Nanjing, Jiangsu 210009, China; ^6^Department of Epidemiology and Biostatistics, School of Public Health, Nantong University, Nantong, Jiangsu 226000, China; ^7^Key Lab of Reproduction Regulation of NPFPC, SIPPR, IRD, Fudan university, Shanghai 200237, China

## Abstract

**Objectives:**

AQP7 and AQP9 represent glycerol channel in adipose tissue and liver and have been associated with metabolic diseases. We aimed to investigate the associations between genetic variants in* AQP7* and* AQP9* genes and the risk of type 2 diabetes (T2DM) in Chinese population.

**Methods:**

Blood samples were drawn from 400 T2DM patients and 400 age- and gender-matched controls. Genomic DNA was extracted by proteinase K digestion and phenol–chloroform extraction. Genotyping of 5 single nucleotide polymorphisms (SNPs) in AQP7 (rs2989924, rs3758269, and rs62542743) and AQP9 (rs57139208, rs16939881) was performed by the polymerase chain reaction assay with TaqMan probes.

**Results:**

The subjects with rs2989924 GA+AA genotypes had 1.47-fold increased risk of T2DM (odds ratio [OR] 1.47, 95% confidence interval [CI] 1.06-2.04), compared to those with GG genotype, and this association remained significant after adjustment for covariates (OR 1.66, 95% CI 1.07-2.57). When compared with rs3758269 CC genotype, the subjects with CT+TT genotypes had 45% decreased T2DM risk after multivariate adjustment (OR 0.55, 95% CI 0.35-0.85). The associations were evident in elder and overweight subjects and those with central obesity. No association was observed between* AQP9 *SNPs and T2DM risk.

**Conclusions:**

* AQP7* SNP rs2989924 and rs3758269 were associated with T2DM risk in Chinese Han population.

## 1. Introduction

The last three decades have witnessed an epidemic rise in the number of people with diabetes, especially type 2 diabetes (T2DM), which accounts for more than 90% of the patients [1, 2]. One-third of the people with diabetes live in China, and the overall prevalence of diabetes in Chinese adults almost doubled in a decade [1, 3, 4]. The causes of the epidemic of T2DM are embedded in a very complex group of genetic and epigenetic systems interacting within an equally complex societal framework that determines behavior and environmental influences [5]. Disparity in the risk of T2DM between different ethnic groups after controlling for diverse environmental attributes indicates a genetic predisposition in the development of T2DM [5]. Although genome-wide association studies in various ethnic groups including Chinese population have identified more than 80 T2DM susceptibility loci, such as genetic variants in* TCF7L2* gene [6–10], these susceptibility loci identified so far can only explain around 10% of the heritability [2, 7, 10]. Therefore, a substantial amount of additional work is still required to identify causal variants/genes and molecular mechanisms via which the association signals found confer diabetes risk [11].

Aquaglyceroporin 7 (AQP7) and AQP9 are transmembrane proteins that permeabilize glycerol as well as water [12–16]. Adipocyte is a major source of glycerol, which is one of the substrates for hepatic gluconeogenesis [16]. AQP7, the adipose-specific glycerol channel, represents a gateway for the delivery of adipose-derived glycerol into plasma [15, 17, 18]. AQP7 deficiency has been linked to triglycerides (TG) accumulation in adipose tissue, adipocyte hypertrophy, adult onset obesity, and insulin resistance [18–20]. The human* AQP7* gene is localized in a chromosomal region with reported linkage to T2DM [21] and the metabolic syndrome [22]. Prudente et al. [23] were the first group to describe single nucleotide polymorphisms (SNPs) in* AQP7* gene and reported that A-953G (rs2989924) in the promoter of* AQP7* was associated with obesity and T2DM in Caucasians. Another genetic variant in* AQP7* gene, named G264V (rs62542743), although was reported to be related to an impaired exercise-induced plasma glycerol increase [24], neither associated with obesity nor associated with diabetes was identified [25].

AQP9 expressed in liver facilitates the hepatic uptake of plasma glycerol and thereby the availability of glycerol for* de novo* synthesis of glucose and TG that both are involved in the pathophysiology of diabetes [15, 17, 18]. However, thus far, there is a lack in association studies of* AQP9* gene and metabolic disorders. For better understanding the genetic predisposition so as to help shape prevention programs and tailor the management of T2DM patients, we conducted a population-based case-control study to explore the associations between* AQP7* and* AQP9* genetic variants and T2DM risk in Chinese.

## 2. Methods

### 2.1. Study Population

The population in this case-control study was recruited from the individuals who participated in a community-based surveillance of T2DM prevalence which was conducted in 2014 in Nanjing, China. The cases were diagnosed as having T2DM according to the American Diabetes Association criteria [26]. We excluded the patients who (1) had type 1 or other types of diabetes such as gestational diabetes mellitus; (2) had malnutrition (body mass index [BMI] < 18.5 kg/m^2^); (3) were obese due to diseases (e.g., islet cell tumor, Cushing's syndrome, polycystic ovarian syndrome, hypogonadism, and hypothyroidism) or medicines (e.g., glucocorticoid, oral contraception); (4) were cancer patients, pregnant women, the disabled, or mentally disturbed. The controls had no history of diabetes mellitus or metabolic diseases and were frequency-matched with the cases according to age, gender, and residential area. All the enrolled subjects were genetically unrelated Han Chinese adults. This study was approved by the Institutional Review Board of Jiangsu Provincial Center for Disease Control and Prevention and each participant signed a written informed consent.

### 2.2. Data Collection

The participants underwent a face-to-face interview by trained interviewers to complete a structured questionnaire including demographic data, lifestyle information, medical history, and family history. After interview, anthropometric index (height, body weight, and waist circumference) and blood pressure were measured. Five milliliters of venous blood was drawn from each subject after an overnight fasting. Then serum was extracted for lipid tests and blood clot for genotyping assays. Methods of measurement of anthropometric index, blood pressure, and serum lipids were described previously [27]. BMI was calculated as weight (kg)/height (m^2^). An individual was overweight when having a BMI ≥ 24 kg/m^2^, and central obesity was defined according to the waist circumference of the participant [4].

### 2.3. SNP Selection and Genotyping Assay

Genomic DNA was extracted by proteinase K digestion and phenol–chloroform extraction [28]. Five SNPs (rs2989924, rs3758269, and rs62542743 in* AQP7*; rs57139208 and rs16939881 in* AQP9*) were selected as candidates because they were (1) potentially functional (nonsynonymous variants or variants in promoter region/5'-UTR/3'-UTR) or significantly associated with T2DM/obesity in previous reports; (2) with a minor allele frequency ≥ 5% in Han Chinese population. Genotyping of these SNPs was performed by the polymerase chain reaction assay with TaqMan probes in ABI Prism® 7900HT Sequence Detection System (Applied Biosystems, Foster City, CA, USA) according to the manufacturer's instructions [28]. Allelic discrimination was automatically completed using the Sequence Detection Systems 2.3 software. The genotyping call rates for 5 SNPs were all > 97% in this study. For quality control, the DNA samples from approximately equal numbers of cases and controls and two blank controls were run in the same batch, and the operators performing detection and genotyping did not know the case or control status of each sample. Moreover, 10% of genotyping samples were randomly chosen to redo the assay blindly for consistency check.

### 2.4. Statistical Analysis

The database was set up by EpiData 3.1 software and analyzed using SPSS 19.0 software (SPSS Inc., Chicago, IL, USA). The differences in demographic characteristics and selected variables between cases and controls were compared using a Student *t*-test for continuous variables and a *χ*^2^ test for categorical variables. The Hardy-Weinberg equilibrium was tested by goodness-of-fit *χ*^2^ test to compare the observed genotype frequencies to the expected ones among the controls. The associations between the genotypes and risk of T2DM were evaluated using univariate and multivariate logistic regression analyses to determine the unadjusted and adjusted odds ratio (OR) and 95 % confidence interval (CI). All statistical tests were two-sided. A* P* value of < 0.05 was considered as statistically significant.

## 3. Results

### 3.1. Demographic Characteristics of the Study Population

A total of 400 T2DM patients and 400 controls were included in this study. The cases and controls were comparable in age, the proportions of males, central obesity and overweight, and the levels of diastolic blood pressure and high density lipoprotein cholesterol (Table 1). Compared with controls, T2DM patients had significantly higher systolic blood pressure (*P* = 0.022), fasting glucose (*P* < 0.001), total cholesterol (*P* < 0.001), TG (*P* < 0.001), and low density lipoprotein cholesterol (*P* < 0.001) (Table 1).

### 3.2. Associations between* AQP* Polymorphisms and T2DM Risk

All the 5 SNPs conformed to Hardy-Weinberg equilibrium. As shown in Table 2, the subjects with rs2989924 GA+AA genotypes had 1.47-fold increased risk of T2DM (OR 1.47, 95% CI 1.06-2.04), compared to those with GG genotype, and this association remained significant after adjustment for covariates (OR 1.66, 95% CI 1.07-2.57). When compared with rs3758269 CC genotype, the subjects with CT+TT genotypes had 45% decreased T2DM risk after multivariate adjustment (OR 0.55, 95% CI 0.35-0.85). No association was observed for SNPs rs62542743, rs57139208, and rs16939881 (Table 2).

### 3.3. Stratified Analyses of Associations of SNPs rs2989924 and rs3758269

Table 3 shows the results of stratified analyses. Significantly increased risk of T2DM in rs2989924 GA+AA genotypes was observed in elder subjects (OR 1.67, 95 % CI 1.02-2.72), overweight subjects (OR 1.88, 95% CI 1.02-3.44), and those with central obesity (OR 1.92, 95% CI 1.11-3.34). Similarly, the association between rs3758269 and T2DM risk was evident in elder subjects (OR 0.50, 95% CI 0.30-0.84), overweight subjects (OR 0.49, 95% CI 0.27-0.89), subjects with central obesity (OR 0.60, 95% CI 0.38-0.95), and females (OR 0.28, 95% CI 0.13-0.59).

## 4. Discussion

In this population-based case-control study, we investigated 5 SNPs in* AQP7* and* AQP9* genes and demonstrated that SNPs rs2989924 and rs3758269 in* AQP7* gene were independently associated with the risk of T2DM. However, no association was observed between* AQP9* SNPs and T2DM risk.

Glycerol constitutes an important metabolite as a substrate for* de novo *synthesis of triacylglycerols and glucose as well as energy substrate to produce ATP; thus the control of glycerol influx/efflux in metabolic organs by AQPs plays a crucial role with the dysregulation of these glycerol channels being associated with metabolic diseases [29]. Physiological and pathological coordinated regulation of organ-specific glycerol channels, adipose AQP7, and liver AQP9 is crucial to determine glycerol metabolism, glucose homeostasis, and insulin resistance* in vivo* [13, 15, 17].

The insulin response element (IRE) and peroxisome proliferator response element have been identified in the promoter region of* AQP7* gene [16, 17, 24, 30], and, thus, adipose AQP7 expression is sensitive to fasting/refeeding and plasma insulin level [14, 17, 18]. Prudente et al. reported that SNP rs2989924 (A-953G) located in the promoter of* AQP7* gene was associated with obesity and secondary development of T2DM in Caucasians, and they further demonstrated this SNP to be functional because it caused decreased transcriptional activity by impairing C/EBP*β* binding to the promoter region and was coupled to decreased AQP7 mRNA abundance in adipose tissue [23]. Our current study, although conducted in the population with different ethnic origin, consistently demonstrated the increased T2DM risk in rs2989924 variant G allele carriers.

Previous study identified a putative IRE in the promoter region of AQP9 as well [17]. The hepatic expression of AQP9, like AQP7 in adipose tissue, is inversely regulated by insulin. PPAR*α* has also been linked to regulation of hepatic AQP9 abundance [18]. Significant reduction of liver AQP9 mRNA levels was reported in obese T2DM woman [31]. However, no study so far, investigates the association of human AQP9 gene and metabolic disorders. In the current study, we did not identify an association between* AQP9 *SNPs and T2DM risk. Although many studies demonstrated that rat AQP9 permeates water, glycerol, and urea, in the study of Ishibashi et al., AQP9-induced permeability was restricted to water and urea in human [32]. Future studies are needed to clarify whether the failure in identification of associations between AQP9 and T2DM risk in human beings is due to species difference in AQP9-induced permeability.

In Prudente's study, the association of rs2989924 was only observed in females, but not in males [23]. Thus, the role of AQP7 in adipose tissue metabolism seemed to be influenced by gender, and it indicated that in women a high AQP7 expression level in adipose tissue is desirable [18]. The assumption of gender-specific effect was supported by the higher abundance of AQP7 in white adipose tissue found in females than in males [33, 34]. The higher need for AQP7 to support glycerol efflux from adipose tissue in women seems well related to the higher plasma glycerol level found in women in response to metabolic stress such as fasting and exercise [18]. However, in our study, we did not identify a gender-specific association of SNP rs2989924 after multivariate adjustment, suggesting that this genetic variant may not be an independent risk factor for T2DM in both genders. Interestingly, we found that the associations of two AQP7 SNPs were evident in the subjects who were overweight or with central obesity (Table 3). It was reported that Asian women carried greater abdominal and visceral adipose when compared with Caucasian women with similar overall adiposity, which may contribute to elevated metabolic risk for obesity-related diseases [35]. Therefore, it is worthwhile of investigating whether genetic variants of obesity and metabolic diseases reported in Caucasians could be generalized to Asians.

In a word, our study demonstrated that the genetic variants of AQP7 SNPs were associated with T2DM risk in Chinese. Further external validations using large and independent samples are warranted to confirm our findings, and future functional studies of associated SNPs will help to better understand the underlying biological mechanisms.

## Figures and Tables

**Table 1:** Demographic and clinical characteristics in diabetes cases and controls.

**Variables**	**Diabetes**	**Controls**	***P***
	**mean ± SD**	**mean ± SD**	
Age (years)	58.08 ± 8.45	57.00 ± 8.30	0.069
Males	209 (52.3)^*∗*^	209 (52.3)^*∗*^	1.00
Overweight	238 (59.5)^*∗*^	241 (60.1)^*∗*^	0.829
Central obesity	272 (68.3)^*∗*^	282 (71.6)^*∗*^	0.32
Systolic blood pressure (mmHg)	148.60 ± 24.67	145.14 ± 17.47	0.022
Diastolic blood pressure (mmHg)	85.51 ± 14.50	84.01 ± 12.10	0.113
Fasting glucose (mmol/L)	8.96 ± 2.71	5.16 ± 1.08	<0.001
Total cholesterol (mmol/L)	5.28 ± 1.09	4.52 ± 0.85	<0.001
Triglycerides (mmol/L)	2.27 ± 2.24	1.66 ± 1.04	<0.001
High density lipoprotein cholesterol (mmol/L)	1.39 ± 0.42	1.43 ± 0.38	0.101
Low density lipoprotein cholesterol (mmol/L)	2.97 ± 0.89	2.20 ± 0.73	<0.001

SD, standard deviation.

^*∗*^represents N (%).

**Table 2:** Genotype frequency distribution in diabetes cases and controls.

**Variable**	**Diabetes, N (%)**	**Control, N (%)**	**χ** ^2^	***P***	**OR**	**95% CI**	**OR** **∗**	**95% CI** **∗**
**rs2989924**								
AA	83 (23.4)	116 (30.9)	7.55	0.02	1.00		1.00	
AG	207 (58.3)	182 (48.5)			1.59	1.13-2.25	1.32	0.89-1.95
GG	65 (18.3)	77 (20.5)			1.18	0.76-1.82	1.12	0.87-1.43
AA	83 (23.4)	116 (30.9)	5.25	0.022	1.00		1.00	
AG+GG	272 (76.6)	259 (69.1)			1.47	1.06-2.04	1.66	1.07-2.57
AA+AG	290 (81.7)	298 (79.5)	0.58	0.45	1.00		1.00	
GG	65 (18.3)	77 (20.5)			0.87	0.60-1.25	0.87	0.55-1.36
**rs3758269**								
CC	291 (73.3)	270 (68.5)	2.19	0.34	1.00		1.00	
CT	96 (24.2)	112 (28.4)			0.79	0.58-1.09	0.62	0.43-0.90
TT	10 (2.5)	12 (3.1)			0.77	0.33-1.82	0.88	0.52-1.49
CC	291 (73.3)	270 (68.5)	2.18	0.14	1.00		1.00	
CT+TT	106 (26.7)	124 (31.5)			0.79	0.58-1.08	0.55	0.35-0.85
CC+CT	387 (97.5)	382 (97.0)	0.20	0.65	1.00		1.00	
TT	10 (2.5)	12 (3.0)			0.82	0.35-1.93	1.25	0.40-3.93
**rs62542743**								
CC	378 (95.7)	363 (92.8)	3.96	0.14	1.00		1.00	
CA	17 (4.3)	27 (6.9)			0.61	0.32-1.13	0.70	0.35-1.41
AA	0 (0.0)	1 (0.3)			0.997	0.99-1.00	-	-
CC	378 (95.7)	363 (92.8)	2.97	0.09	1.00		1.00	
CA+AA	17 (4.3)	28 (7.2)			0.58	0.31-1.08	0.88	0.41-1.89
CC+CA	395 (100.0)	390 (99.5)	1.40	0.24	1.00		1.00	
AA	0 (0.00)	2 (0.5)			0.99	0.99-1.01	-	-
**rs57139208**								
CC	299 (75.3)	292 (74.5)	0.59	0.75	1.00		1.00	
CT	91 (22.9)	90 (23.0)			0.99	0.71-1.38	0.83	0.56-1.23
TT	7 (1.8)	10 (2.5)			0.68	0.26-1.82	0.77	0.44-1.35
CC	299 (75.3)	292 (74.5)	0.07	0.79	1.00		1.00	
CT+TT	98 (24.7)	100 (25.5)			0.96	0.69-1.32	0.83	0.54-1.27
CC+CT	390 (98.2)	382 (97.4)	0.58	0.45	1.00		1.00	
TT	7 (1.8)	10 (2.6)			0.69	0.26-1.82	0.56	0.15-2.03
**rs16939881**								
GG	345 (86.9)	331 (87.1)	1.53	0.47	1.00		1.00	
GC	45 (11.4)	46 (12.1)			0.94	0.61-1.45	0.99	0.60-1.65
CC	7 (1.8)	3 (0.8)			2.24	0.57-8.73	1.65	0.76-3.55
GG	345 (86.9)	331 (87.1)	0.01	0.93	1.00		1.00	
GC+CC	52 (13.1)	49 (12.9)			1.02	0.67-1.55	1.06	0.62-1.84
GG+GC	390 (98.2)	377 (99.2)	1.45	0.23	1.00		1.00	
CC	7 (1.8)	3 (0.8)			2.26	0.58-8.79	2.50	0.37-17.02

OR, odds ratio; CI, confidence interval.

*∗* Adjusted for age, gender, systolic blood pressure, fasting glucose, total cholesterol, triglycerides, and low density lipoprotein cholesterol.

**Table 3:** Stratified analysis between SNPs and type 2 diabetes.

	**Additive model**						**OR (95**%** CI)∗**	**Recessive model**				**OR (95**%** CI)∗**	**Dominant model**				**OR (95**%** CI)∗**
	**Diabetes**			**Controls**				**Diabetes**		**Controls**			**Diabetes**		**Controls**		
	**DD (**%**)**	**Dd (**%**)**	**dd (**%**)**	**DD (**%**)**	**Dd (**%**)**	**dd (**%**)**		**DD+Dd (**%**)**	**dd (**%**)**	**DD+Dd (**%**)**	**dd (**%**)**		**DD (**%**)**	**Dd+dd (**%**)**	**DD (**%**)**	**Dd+dd (**%**)**	
**rs2989924**																	
Age ≤ 50 (years)	12 (18.5)	38 (58.5)	15 (23.1)	23 (28.0)	41 (50.0)	18 (22.0)	1.46 (0.86-2.47)	50 (76.9)	15 (23.1)	64 (78.0)	18 (22.0)	1.12 (0.46-2.73)	12 (18.5)	53 (81.5)	23 (28.0)	59 (72.0)	1.91 (0.77-4.76)
Age > 50 (years)	71 (24.5)	169 (58.3)	50 (17.2)	93 (31.7)	141 (48.1)	59 (20.1)	1.67 (1.02-2.72)	240 (82.8)	50 (17.2)	234 (79.9)	59 (20.1)	0.83 (0.54-1.25)	71 (24.5)	219 (75.5)	93 (31.7)	200 (68.3)	1.67 (1.02-2.72)
Females	47 (26.3)	106 (59.2)	26 (14.5)	57 (31.0)	95 (51.6)	32 (17.4)	0.98 (0.67-1.42)	153 (85.5)	26 (14.5)	152 (82.6)	32 (17.4)	0.73 (0.36-1.51)	47 (26.3)	132 (73.7)	57 (31.0)	127 (69.0)	1.19 (0.67-2.12)
Males	36 (20.5)	101 (57.4)	39 (22.2)	59 (30.9)	87 (45.5)	45 (23.6)	1.21 (0.87-1.02)	137 (77.8)	39 (22.2)	146 (76.4)	45 (23.6)	0.94 (0.53-1.66)	36 (20.5)	140 (79.5)	59 (30.9)	132 (69.1)	1.54 (0.88-2.70)
Normal weight	35 (24.3)	75 (52.1)	34 (23.6)	48 (33.1)	72 (49.7)	25 (17.2)	1.40 (0.96-2.04)	110 (76.4)	34 (23.6)	120 (82.8)	25 (17.2)	1.76 (0.92-3.39)	35 (24.3)	109 (75.7)	48 (33.1)	97 (66.9)	1.41 (0.79-2.52)
Overweight	48 (22.7)	132 (62.6)	31 (14.7)	68 (29.6)	110 (47.8)	52 (22.6)	1.16 (0.85-1.59)	180 (85.3)	31 (14.7)	178 (77.4)	52 (22.6)	1.49 (0.98-2.25)	48 (22.7)	163 (77.3)	68 (29.6)	162 (70.4)	1.88 (1.02-3.44)
Without central obesity	28 (25.0)	61 (54.5)	23 (21.2)	31 (29.8)	51 (49.0)	22 (21.2)	1.01 (0.65-1.58)	89 (79.5)	23 (20.5)	82 (78.8)	22 (21.2)	1.08 (0.50-2.35)	28 (25.0)	84 (75.0)	31 (29.8)	73 (70.2)	0.95 (0.46-1.99)
With central obesity	55 (22.7)	146 (60.3)	41 (16.9)	84 (31.7)	126 (47.5)	55 (20.8)	1.30 (0.98-1.72)	201 (83.1)	41 (16.9)	210 (79.2)	55 (20.8)	0.78 (0.49-1.22)	55 (22.7)	187 (77.3)	84 (31.7)	181 (68.3)	1.92 (1.11-3.34)
**rs3758269**																	
Age ≤ 50 (years)	57 (74.0)	16 (20.8)	4 (5.2)	63 (70.8)	26 (29.2)	0 (0.0)	0.93 (0.48-1.81)	73 (94.8)	4 (5.2)	89 (100.0)	0 (0.0)	-	57 (74.0)	20 (26.0)	63 (70.8)	26 (29.2)	0.62 (0.28-1.35)
Age > 50 (years)	234 (73.1)	80 (25.0)	6 (1.9)	207 (67.9)	86 (28.2)	12 (3.9)	0.56 (0.36-0.86)	314 (98.1)	6 (1.9)	293 (96.1)	12 (3.9)	0.47 (0.17-1.26)	234 (73.1)	86 (26.9)	207 (67.9)	98 (32.1)	0.50 (0.30-0.84)
Females	146 (76.4)	41 (21.5)	4 (2.1)	134 (71.3)	50 (26.6)	4 (2.1)	0.44 (0.24-0.81)	187 (97.9)	4 (2.1)	184 (97.9)	4 (2.1)	0.98 (0.24-3.99)	146 (76.4)	45 (23.6)	134 (71.3)	54 (28.7)	0.28 (0.13-0.59)
Males	145 (70.4)	55 (26.7)	6 (2.9)	136 (66.0)	62 (30.1)	8 (3.9)	0.79 (0.53-1.19)	200 (97.1)	6 (2.9)	198 (96.1)	8 (3.9)	0.71 (0.89-2.67)	145 (70.4)	61 (29.6)	136 (66.0)	70 (34.0)	0.81 (0.49-1.31)
Normal weight	117 (72.7)	40 (24.8)	4 (2.5)	111 (70.7)	43 (27.4)	3 (1.9)	0.83 (0.48-1.45)	157 (97.5)	4 (2.5)	154 (98.1)	3 (1.9)	1.21 (0.21-7.08)	117 (72.7)	44 (27.3)	111 (70.7)	46 (29.3)	0.75 (0.42-1.34)
Overweight	174 (73.7)	56 (23.7)	6 (2.5)	159 (67.1)	69 (29.1)	9 (3.8)	0.59 (0.35-0.99)	230 (97.5)	6 (2.5)	228 (96.2)	9 (3.8)	0.66 (0.23-1.88)	174 (73.7)	62 (26.3)	159 (67.1)	78 (32.9)	0.49 (0.27-0.89)
Without central obesity	95 (76.0)	27 (21.6)	3 (2.4)	77 (69.4)	31 (27.9)	3 (2.7)	0.62 (0.35-1.10)	122 (97.6)	3 (2.4)	108 (97.3)	3 (2.7)	1.26 (0.19-8.13)	95 (76.0)	30 (24.0)	77 (69.4)	34 (30.6)	0.53 (0.26-1.06)
With central obesity	195 (72.2)	68 (25.2)	7 (2.6)	188 (67.9)	80 (28.9)	9 (3.2)	0.65 (0.44-0.98)	263 (97.4)	7 (2.6)	268 (96.8)	9 (3.2)	1.10 (0.26-4.63)	195 (72.2)	75 (27.8)	188 (67.9)	89 (32.1)	0.60 (0.38-0.95)

OR, odds ratio; CI, confidence interval; D, wild-type allele; d, risk allele; BMI, body mass index.

*∗* Adjusted for age, gender, systolic blood pressure, fasting glucose, total cholesterol, triglycerides, and low density lipoprotein cholesterol, where appropriate.

## Data Availability

The data used to support the findings of this study are available from the corresponding author upon request.
